# Personals

**Published:** 1914-04

**Authors:** 


### PERSONALS

---

***Announcement of removal, travel, and other matters of interest are requested. Please report errors in the listing of any physician in the State and other directories that we may co-operate with the State Society in securing a correct list.***

---

Dr. Thew Wright of Buffalo returned March 13 from a trip to South America, Panama and Cuba.

---

Dr. D. H. Arthur, Superintendent of the Gowanda State Hospital, resigned March 12.

---

On February 20, by invitation, Drs. E. W. Saunders of St. Louis, Roland Meisenbach of Buffalo and W. E. Wisdom of Arkansas presented their work on Infantile Paralysis before the University of Virginia, in Richmond.

---

Dr. Edward Meyer of Buffalo visited French Lick Springs in March.

Dr. Clayton M. Browne of Buffalo has bought the residence of the late Dr. Roswell Park.

---

Dr. Henry Adsit took a short trip to Baltimore and New York late in February.

---

Dr. Walter V. Girvin of Jamestown returned early in March from a western trip of several weeks.

---

Dr. Regina Flood Keyes of Buffalo took the Panama-west Indies trip in February.

---

Dr. M. D. Mann of Buffalo has returned from a trip to California.

---

Dr. F. M. Evans has been elected President of the village of Fredonia.

---

Dr. Charles W. Banta of Buffalo returned about the middle of March from a trip to Bermuda, stopping for a week in Baltimore to study Dr. Howard Kelly's work.

---

Dr. N. G. Richmond of Fredonia is spending six weeks hunting and fishing in Florida, returning about the middle of April.

---

Dr. Jane W. Carroll has moved from Buffalo.

---

Dr. W. S. Grimes of Detroit, a University of Buffalo man, has resigned his position as pension examiner on account of pressure of private practice.

---

The editor acknowledges with thanks a complimentary card for the Ragaz-Pfaefers baths, Switzerland. Even a plain, ordinary bath, according to modern American standards of immersion, is often not available in Europe, and we shall not forget a Swiss bath, after several days of wet-rag ablutions, personally conducted by a man servant, a maid servant and a boy, with an imposing array of sheets and towels. We regret that we shall probably not be able to accept this hospitality for another year at least.

At a recent meeting of the Tri-State Medical Society of the Carolinas and Virginia, Dr. E. C. Register, who has been editor of the well-known *Charlotte Medical Journal* for twenty-five years, was elected president.

---

Dr. Ray A. Edson announces the opening of his office at 560 Delaware avenue, Buffalo.

---

Dr. N. Victoria Chappell and Miss E. Mae Chappell, formerly Librarian of the University of Buffalo, have returned from a visit of two months in California.

---

Dr. H. C. Tinkham of Burlington, Dean of the College of Medicine, University of Vermont, and Editor of the *Vt. Med. Monthly*, was in Buffalo March 20 to attend the Alumni dinner, presided over by Hon. Henry W. Hill.

---

Dr. George D. Miller of Warsaw spent the latter part of March in New York.

---

Dr. C. F. Cushing of Niagara Falls will return from Florida about the middle of April.

---

Dr. J. J. Drake of Lackawanna has returned from a two months' trip to California.

